# Mode I Fatigue and Fracture Assessment of Polyimide–Epoxy and Silicon–Epoxy Interfaces in Chip-Package Components

**DOI:** 10.3390/polym16040463

**Published:** 2024-02-07

**Authors:** Pedro Morais, Alireza Akhavan-Safar, Ricardo J. C. Carbas, Eduardo A. S. Marques, Bala Karunamurthy, Lucas F. M. da Silva

**Affiliations:** 1 Faculdade de Engenharia, Universidade do Porto, Rua Dr. Roberto Frias, 4200-465 Porto, Portugal; 2 Institute of Science and Innovation in Mechanical and Industrial Engineering (INEGI), Rua Dr. Roberto Frias, 4200-465 Porto, Portugal; 3 Infineon Technologies Austria AG, Siemensstrasse 2, 9500 Villach, Austria; bala.karunamurthy@infineon.com

**Keywords:** semiconductors, bi-material interface, interfacial fracture, fatigue crack growth, Paris law curve

## Abstract

Semiconductor advancements demand greater integrated circuit density, structural miniaturization, and complex material combinations, resulting in stress concentrations from property mismatches. This study investigates the failure in two types of interfaces found in chip packages: silicon–epoxy mold compound (EMC) and polyimide–EMC. These interfaces were subjected to quasi-static and fatigue loading conditions. Employing a compliance-based beam method, the tests determined interfacial critical fracture energy values, ($G_{IC}$), of 0.051 N/mm and 0.037 N/mm for the silicon–EMC and polyimide–EMC interfaces, respectively. Fatigue testing on the polyimide–epoxy interface revealed a fatigue threshold strain energy, ($G_{th}$), of 0.042 N/mm. We also observed diverse failure modes and discuss potential mechanical failures in multi-layer chip packages. The findings of this study can contribute to the prediction and mitigation of failure modes in the analyzed chip packaging. The obtained threshold energy and crack growth rate provide insights for designing safe lives for bi-material interfaces in chip packaging under cyclic loads. These insights can guide future research directions, emphasizing the improvement of material properties and exploration of the influence of manufacturing parameters on delamination in multilayer semiconductors.

## 1. Introduction

The trend of densifying microelectronic devices for specialized functions has led to complex designs and increased risks, with delamination susceptibility at interfaces involving dissimilar materials. Differences in the coefficient of thermal expansion and Young’s modulus can cause imperfect adhesion and stress concentrations during the manufacturing procedure and in service. These stresses can cause crack initiation and subsequent propagation. As such, the prediction of interfacial failure in these components is very desirable. To determine possible weak points, fracture mechanics concepts can be used to calculate the fracture energy, fatigue threshold energy, and rate of fatigue crack growth. These concepts involve a considerable number of parameters, most notably the loading modes. This study focuses on mode I, which corresponds to tensile stress normal to the place of the crack, with the use of double-cantilever-beam (DCB) joints.

Over the years, numerous material interfaces in the chip packaging industry have been studied. One of the most extensively researched interactions is between epoxy molding compound (EMC) and copper (Cu). Samet [1,2] focused on delamination in EMC–Cu interfaces under fatigue loading by using DCB, four-point-bending (4PB), and dissimilar-mixed-mode-bending (DMMB) tests. The slope of the Paris law curve *m* showed a clear dependency on mode mixity. Using crowbar loading (CBL), Rambhatla and Sitaraman [3] characterized the Cu–EMC interface under quasi-static loading. The mode I fracture energy ($G_{IC}$) obtained for CBL was 0.123 N/mm higher than that obtained for the DCB test (0.060 N/mm). The use of the DCB test as a standard test method for mode I fracture tests was also explored by Calabretta et al. [4], who obtained a $G_{IC}$ of 0.044 N/mm for the same interface. Geers et al. [5] found lower interface strength in mixed-mode loading compared to DCB joints under pure mode I loading, challenging the DCB test’s suitability as a worst-case test in this study. A comprehensive literature review revealed extensive research on the Cu–EMC interface, especially in characterizing it under various mode mixities.

Other types of interfaces seen in the literature are silicon nitride–Cu, polyimide–silicon nitride, silicon–polyimide, and polyimide–gold. A study on the fracture behavior and crack propagation of silicon nitride–Cu interfaces was undertaken by Yan et al. [6] with a modified DCB test. The critical energy release rate for this interface was 0.0021 N/mm for quasi-static mode-I-dominated conditions. Polyimide–silicon nitride, polyimide–silicon, and polyimide–gold interfaces were tested using a DCB setup under monotonic loading conditions by Zhu et al. [7]. The polyimide–silicon nitride interface was also tested under fatigue loading conditions. The critical energy release rate for polyimide–silicon nitride was 0.139 N/mm higher than the energy obtained for polyimide–silicon (0.041 N/mm) and polyimide–gold (0.007 N/mm). The $G_{th}$ value for polyimide–silicon nitride was 0.015 N/mm and *m* was 3.2. The higher toughness when comparing polyimide–silicon nitride and polyimide–silicon may be attributed to the higher roughness of the silicon nitride surface, which results in better mechanical bonding to the polyimide thin film.

Despite the extensive studies, however, the fatigue and fracture of silicon–EMC and EMC–polyimide interfaces have not been explored in the literature. Despite silicon’s well-established reputation for offering robust mechanical and electrical characteristics, its inherent brittleness poses challenges. In contrast, EMC has been widely utilized to safeguard materials with such characteristics thanks to its flexible properties. Schlottig [8] explained that the scarcity of studies on this specific interface can be attributed to the inherent challenges associated with delaminating it. During the manufacturing of these wafers, EMC and silicon are joined at high temperatures (usually at 175 °C), and during curing and cooling-down processes, these materials warp, creating residual stresses that should be accounted for. Wang et al. [9] experimentally characterized this interface in terms of fracture toughness. However, ($G_{IC}$) was only calculated under thermal loading conditions, which led to interfacial delamination.

Given the documented challenges found in the literature regarding the interplay between silicon and EMC and the limited information available on the existence of thin-film materials between silicon and EMC, our current research aimed to fill this gap. We addressed these issues by conducting both quasi-static and fatigue fracture tests. We performed mode I static and fatigue fracture tests at room temperature using DCB joints. Although our main focus lay in characterizing the silicon–EMC bi-material interface, it is important to note that failures do not occur exclusively at the interface. Various factors, such as loading conditions and the properties of silicon and EMC materials, can impact crack propagation. As a result, our study also explored failures within the silicon layer and mixed failures involving both the silicon layer and the bi-material interface.

## 2. Experimental Section

### 2.1. Materials

**Figure 1:**
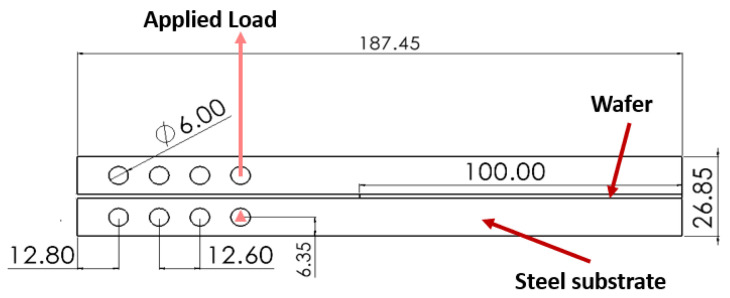
Technical drawing of DCB joint and wafer. Dimensions in mm.

Two types of wafers were examined, primarily composed of silicon and EMC and featuring a pre-manufactured gold pre-crack layer. The wafers were divided into two types: type one (silicon–epoxy mold compound (EMC)) and type two (polyimide–EMC). Both types of wafers were already pre-manufactured with a built-in gold pre-crack, as shown in Figure 1. The difference between these two types of interfaces was the existence of two extra material layers between the silicon and EMC: polyimide and silicon oxide were added to the type-two interface. EMC is widely used in packaging semiconductor devices, underscoring its critical role in influencing the structural integrity of semiconductor components. It is a flexible glass-fiber-reinforced epoxy made from epoxy resin, hardener, silica, and other additives, with an undisclosed weight percentage for each component. The additives incorporated into EMC are intended to improve its mechanical and physical properties. EMC varies in terms of the density of the fibers, length, and composition and effectively protects semiconductor circuits from heat, shock, and moisture [10]. In contrast to EMC, silicon is more brittle. It is the most-used material in semiconductors and is usually found in these components as a single-crystal structure of ultra-high purity [11]. Polyimide is a high-performance polymer of imide monomers. Commonly used as a dielectric in semiconductors, polyimide has high thermal stability and excellent chemical resistance and electrical insulation properties, making it ideal for creating insulating layers [12]. Silicon oxide acts as a prevalent buffer layer in semiconductors. A silicon oxide layer of just a few nm in thickness acts as an efficient diffusion barrier, preventing the diffusion of silicon into gold [13]. It is essential to note that the EMC has a thickness in the range of a few hundred microns, whereas the silicon oxide layer, as mentioned above, is merely a few nanometers thick. PI and silicon oxide are pure materials, whereas EMC is a composite material, as mentioned above. The properties of silicon, EMC, polyimide, silicon oxide, and PM300 are represented in Table 1.

Steel substrates (PM300) were utilized to support the wafers during testing, securely bonded using a structural adhesive. PM300 is a high-strength steel and as such presents much higher strength and stiffness than the other materials and suffers less deformation. Since the objective of this investigation was to study the characteristics of bi-material interfaces, it was crucial to ensure that the selected adhesive could withstand the testing loads without failure. With this requisite in mind, adhesive AV138 M1 and its corresponding hardener HV 998-1 were chosen. This is a very stiff and brittle epoxy used in aerospace applications with the properties shown in Table 2. The resin-to-hardener ratio considered for the adhesive was 100:40.

#### Specimen Manufacture and Geometry

**Figure 2:**
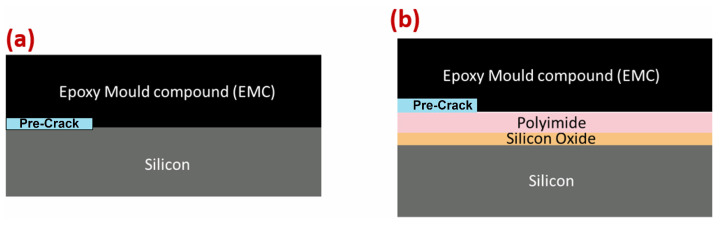
Schematic representation of (**a**) type-one interface and (**b**) type-two interface.

Figure 1 represents a technical drawing of the DCB joint. This joint consists of two steel substrates with a wafer in between. Schematic representations of the two different wafers are shown in Figure 2.

**Figure 3:**
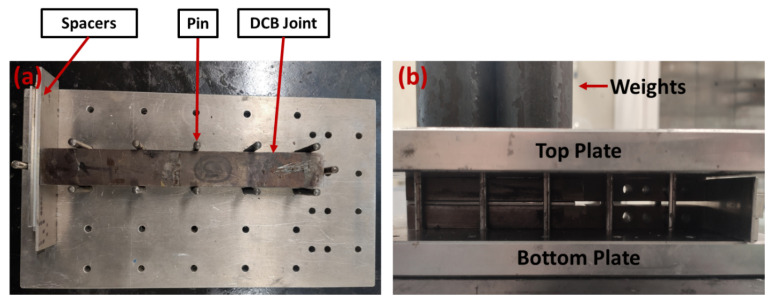
Different views of the DCB joint in the mold. (**a**) Top view and (**b**) curing at room temperature with controlled pressure.

The manufacturing process for DCB joints is initiated by sandblasting both specimen surfaces, eliminating iron oxides and enhancing adhesion. Post-sandblasting, it is critical to remove residual sand using compressed air and to degrease with acetone. The wafer sides undergo abrasion with 800-grit sandpaper at ±45° angles. Subsequently, wafer surfaces are cleaned with acetone, and a 6-second plasma treatment with the Arcotec PG051 machine concludes the process. Abrasion and plasma treatment aim to elevate the surface energy of the wafer surfaces. In the case of EMC, with this treatment surface energy increased from 19.78 mJ/m^2^ to 59.18 mJ/m^2^. The silicon side experienced a rise in surface energy from 30.51 mJ/m^2^ to 39.94 mJ/m^2^. A mold, as illustrated in Figure 3, was employed for DCB manufacturing. Typically, such molds comprise top and bottom plates with guide pin holes to secure the DCBs in position during production. Given the length of the substrates, spacers were essential to ensure a good fit on the mold.

The resin and hardener of the brittle epoxy AV138-HV998 were mixed in a high-speed mixer at a speed of 2500 rpm for 1 min, with a ratio of 1 g of hardener per 2.5 g of resin. The thickness of the applied adhesive was not controlled; however, under the pressure applied to the joint during the curing process, the adhesive was reduced to a very thin layer between the two substrates and the wafer. Following joint assembly, the curing process involved applying controlled pressure to the mold at room temperature for 24 h, as depicted in Figure 3b. Subsequently, the process continued at 60 °C for 2 h without pressure, followed by gradual oven cooling.

### 2.2. Test Procedure

**Figure 4:**
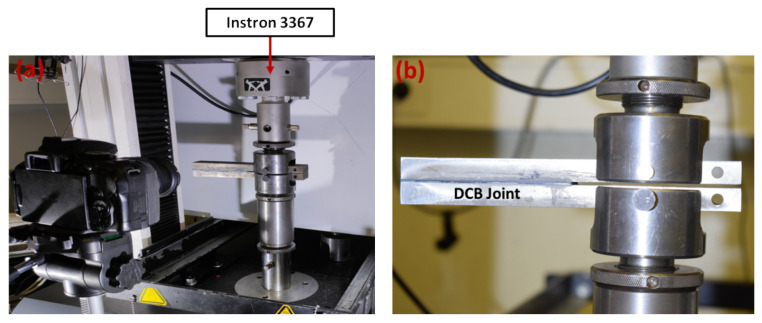
DCB quasi-static test. (**a**) Instron 3367 machine testing setup and (**b**) zoomed-in view of the joint.

The load was applied to the hole nearest to the wafer under room conditions, consistently in mode I. The quasi-static test was conducted using an INSTRON 3367 with a constant displacement rate of 0.2 mm/min. For the fatigue tests, an INSTRON 8801 was employed at a frequency of 1 Hz and an R ratio of 0.33. The maximum fatigue load was determined based on the results initially obtained from the quasi-static tests for each type of interface. The sampling rate for both machines was set to 10 Hz. The test setup is illustrated in Figure 4.

### 2.3. Data Reduction Approach

The machine-generated data were subsequently processed using CBBM to ascertain the mode I fracture energy of the tested sample. This approach calculates the energy release rate for the crack without directly measuring the crack length. Instead, it relies on an equivalent crack length derived from the specimen’s compliance (C). Equation (1) expresses the mode I critical fracture energy ($G_{IC}$):

(1) $$G_{IC}=\frac{6P^{2}}{b^{2}h}\left(\frac{2{\left(a_{eq}\right)}^{2}}{h^{2}E_{f}}+\frac{1}{5G}\right)$$

where *G* is the shear modulus of the adherents and $E_{f}$ is a corrected flexural modulus calculated by Equation (2). $a_{eq}$ represents the equivalent crack length derived from the compliance obtained during experimental testing, where *b* is the width of the specimens, *h* denotes the thickness of the substrate, and *P* represents the applied load. The flexural modulus in Equation (1) is defined as follows:

(2) $$E_{f}={\left(C_{0}-\frac{12(a_{0}+|\Delta |)}{5bhG}\right)}^{-1}\frac{8{\left(a_{0}+\left|\Delta \right|\right)}^{3}}{bh^{3}}$$

The flexural modulus $E_{f}$ is affected by the initial crack length ($a_{0}$), initial compliance ($C_{0}$), and a correction factor for the crack length (Δ). Δ is obtained with Equation (3):

(3) $$\Delta =h\sqrt{\frac{E}{11G}\left(3-2{\left(\frac{\Gamma }{1+\Gamma }\right)}^{2}\right)}\;and\;\Gamma =1.18\frac{E}{G}$$

where *E* and *G* are, respectively, the Young’s modulus and shear modulus of the substrate. The $a_{eq}$ is obtained with Timoshenko’s beam theory using Equation (4), with *C* being the specimen’s compliance obtained from raw data [18]:

(4) $$C=\frac{\delta }{P}=\frac{8{\left(a_{eq}\right)}^{3}}{bh^{3}E_{f}}+\frac{12a_{eq}}{5Gbh}$$

Equations (1)–(4), as mentioned above, show the relationships utilized for processing raw data; specifically, the load line displacements and the respective loads. In each test, considering the rate of data acquisition used, hundreds of data points (displacement, load) were generated, and the equations (Equations (1)–(4)) were applied to calculate the fracture energy and $a_{eq}$ (equivalent crack length) for each data point. Consequently, each test yielded hundreds of values for $G_{Ic}$, $a_{eq}$, *C*, *P*, and δ. $C_{0}$, representing the initial compliance of the specimen (calculated by dividing the displacement by the load for the initial linear segment of the load–displacement curve), is also not a constant value, and each specimen had its own $C_{0}$. All these calculations and the raw data points formed the basis for the curves presented in Section 2.4.1. For other parameters that were dependent solely on geometry or material properties, the values were as follows: *E* and *G* were considered as 210 GPa and 79 GPa, respectively. The initial crack size was set to 65 mm, while *b* (width of the specimen) and *h* (thickness of the substrate) were equal to 25 and 12.7 mm, respectively, considering the geometry shown in Figure 1.

### 2.4. Results and Discussion

#### 2.4.1. Quasi-Static Results

##### Failure Modes

**Figure 5:**
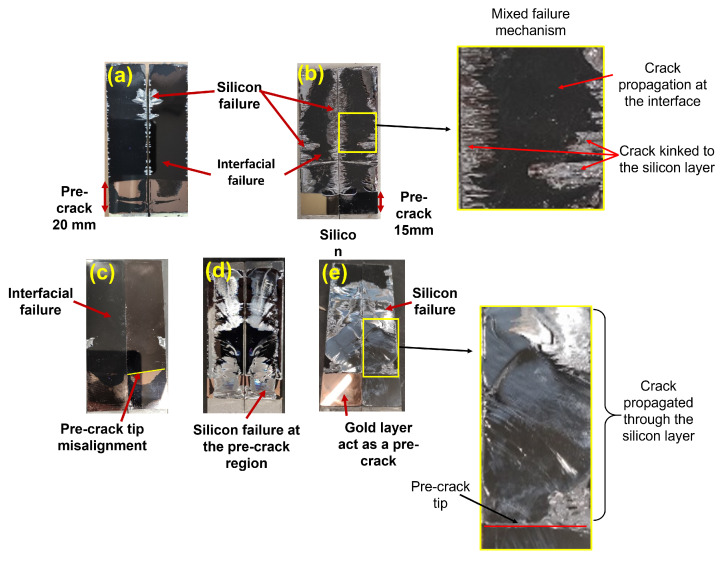
Typical type-one interface: (**a**) interfacial failure and (**b**) mixed failure fracture surfaces. Typical type-two interface: (**c**) interfacial failure, (**d**) mixed failure, and (**e**) silicon failure fracture surfaces. In these surfaces, the dark (black) areas denote interfacial failure, while the shiny (silver-colored) regions signify cracks propagating through the silicon layer.

**Figure 6:**
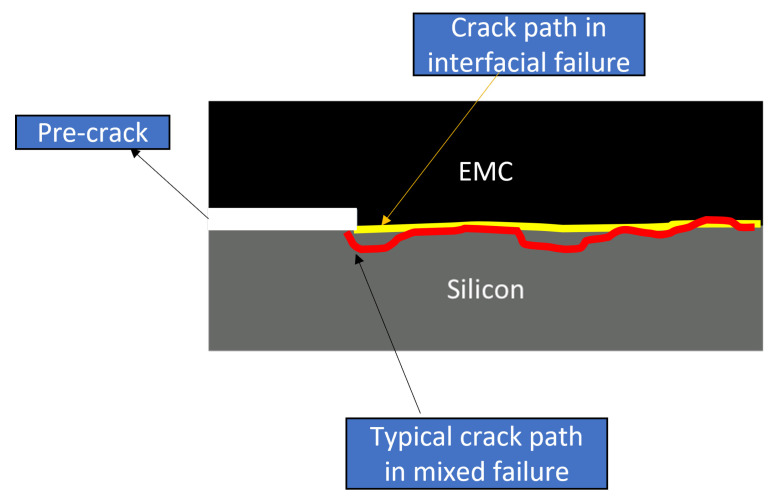
Schematic representation of the crack path for interfacial and mixed failure mechanisms for the type-one specimen.

It is crucial to differentiate and categorize the obtained results based on the observed failure mechanisms for each specimen. In this study, we considered three types of failure modes. First was the interfacial failure, indicating that the crack propagated at the silicon–EMC or polyimide–EMC interface. Another mode was the silicon-layer failure, where the crack propagated through the silicon layer. Lastly, the mixed failure mechanism involved the crack propagating through both the silicon layer and the interface. The weakest section of the joint determines the direction of crack propagation. In the case of the type-one interface, it resulted in interfacial failure and mixed failure. However, for the type-two interface, all three types of failure modes were observed. The typical fracture surfaces of the tested specimens are presented in Figure 5. The crack path is also schematically shown in Figure 6 for the type-one specimen, as well as two different types of failure modes (interfacial failure and mixed failure).

Obtaining the interfacial failure during this study posed a significant challenge. One of the contributing factors was the limitations of conventional testing methods, like DCB, 3PB, 4PB, and mixed-mode bending (MMB), when dealing with brittle and stiff material combinations, such as silicon and EMC. Another obstacle arose from the manufacturing process for the wafers. During production, these two materials were fused at high temperatures and underwent subsequent curing and cooling processes, leading to shrinkage. This shrinkage resulted in the warping of the wafers and the development of residual stresses even before they were subject to quasi-static or cyclic loading conditions. These pre-existing conditions made it more difficult for cracks to propagate through the bi-material interface [8]. It was also common throughout this study to detect micrometer-scale roughness, micro-cracks, and irregularities on the edges of the silicon layer caused during the dicing procedures applied to the wafers to form them into their rectangular shape. The delicate nature of the silicon posed challenges in relation to cutting it into the final rectangle shapes. Irregularities at the edges of the silicon created stress concentrations, diminishing the load-bearing capacity of the silicon layer. Additionally, the quality of the results depended on the production of the pre-crack part, which contributed to substantial variability among otherwise similar results, as observed in the next sections. Various factors during joint manufacturing may also prevent crack propagation through the interface. For instance, excessive weight during the curing process or excess adhesive flowing through joint edges, requiring sandpaper abrasion, can lead to the formation of micro-cracks on the silicon edges. Consequently, the silicon may become weaker than the bi-material interface, causing crack propagation through the weakened edges.

Some of these manufacturing problems can be seen in Figure 5. In Figure 5, the golden portion at the bottom of all the images represents the pre-crack region, the black area signifies EMC, and the silver indicates silicon. Figure 5a reveals various material layers and contamination issues. In Figure 5b, the presence of silicon in the pre-crack region and a shorter pre-crack size are evident. It is noteworthy that, despite a mixed failure, the crack propagated more in the interface than in the silicon due to silicon bonding to the gold layer and the presence of micro-cracks on the edges. Figure 5c highlights misalignment issues, while in the case of Figure 5d, a larger amount of silicon can be observed on the gold layer compared to Figure 5b. Micro-cracks on the edges contributed to the crack propagating more in the silicon layer than at the interface when comparing Figure 5d to Figure 5b. In Figure 5e, no issues were detected in the pre-crack. However, micro-cracks were found on the edges of the wafer, causing a silicon failure for this joint. The variation in the amount of silicon on the fracture surface was attributed to the presence of micro-cracks and irregularities on the wafer edges occurring during wafer or joint manufacturing and bonded pieces of silicon on the gold layer. Depending on the severity of these issues, their impact on crack propagation varies. In some instances, the effect may be less noticeable, allowing most of the crack to propagate through the interface (Figure 5a). Alternatively, the issues can be more substantial, leading to crack propagation in both the interface and silicon (Figure 5b,d). In critical cases, these factors can lead to the crack exclusively advancing in the silicon layer (Figure 5e).

To address irregularities on the edges of silicon layers, a polishing process was employed in this study. Initially, 1200-grit sandpaper was used, followed by a 1 μm size diamond polish. Although polishing contributes to mitigating these manufacturing issues, it is important to note that it does not eliminate them.

##### Fracture Analysis

**Figure 7:**
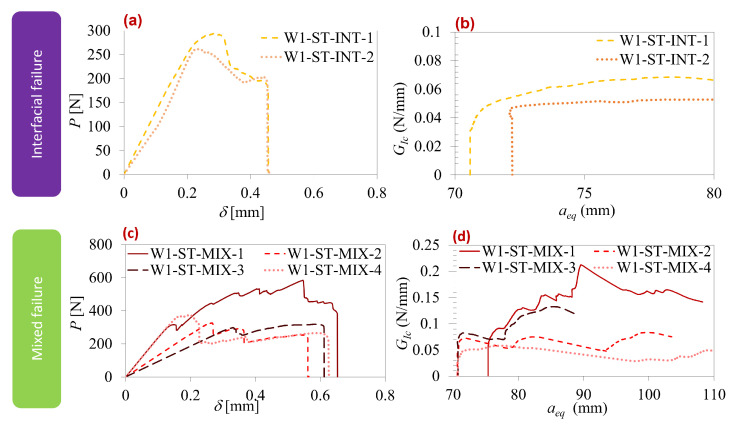
Type-one interface quasi-static results. Interfacial failure: (**a**) load–displacement curve and (**b**) R-curve. Mixed failure: (**c**) load–displacement curve and (**d**) R-curve.

Figure 7 displays the load–displacement curves and R-curves corresponding to each failure mode observed for the type-one interface. Table 3 provides a comparison of the values for maximum load, maximum displacement, and $G_{IC}$ extracted from the graphs in Figure 7. As previously mentioned, the quasi-static type-one interface exhibited only two failure modes: interfacial and mixed. In Figure 7a,b, interfacial failure mode curves showed significant scatter, primarily attributed to varying pre-crack sizes caused by contamination in the pre-crack region. However, both maximum displacement and $G_{IC}$ exhibited similarities, as evident in Figure 7a,b. Regarding the mixed mode, there was an even more substantial discrepancy in the results due to defects in the pre-crack part and varying degrees of silicon edge defects. These factors led to the oscillation of the failure mechanism towards either the interface or the silicon layer. Table 3 indicates that, overall, mixed failure demonstrated superior properties compared to the interfacial failure mode condition. This implies that, when a mixed failure mechanism occurs, the bi-material interfaces are stronger than bonded materials at both sides of the interface.

**Figure 8:**
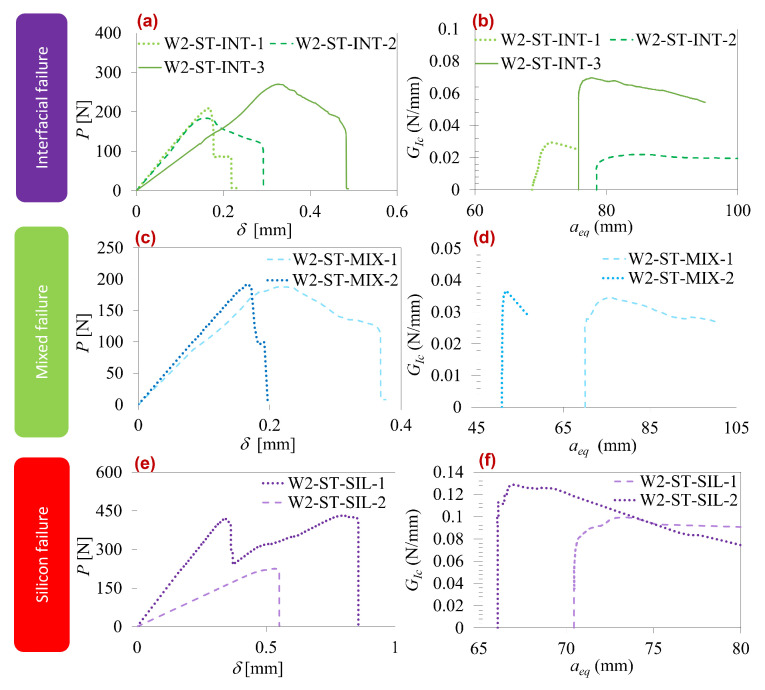
Type-two interface quasi-static results. Interfacial failure: (**a**) load–displacement curve and (**b**) R-curve. Mixed failure: (**c**) load–displacement curve and (**d**) R-curve. Silicon failure: (**e**) load–displacement curve and (**f**) R-curve.

In quasi-static tests, the type-two interface displayed three failure modes, as illustrated in Figure 8: interfacial, mixed, and silicon layer failure. Table 3 compares parameter values for each mode. The interfacial mode exhibited a notable scatter in the curves, particularly in test W2-ST-INT3, where contamination in the pre-crack region caused a more brittle failure at the joint edges. The other two tests showed more aligned behavior. Analyzing Table 3, silicon failure demonstrated higher properties, followed by interfacial failure and, lastly, mixed failure.

Table 3 summarizes the obtained quasi-static results for each interface type and failure mode.

The obtained results indicate that the type-one interface exhibited a higher fracture energy compared to type two. However, it is important to note that, in certain tested components, failure occurred not at the interface but through the silicon layer. Additionally, mixed interface–silicon failures were also observed in some cases. Taking into account the different failure modes and the results shown in Table 3, silicon demonstrated the highest fracture energy.

### 2.5. Fatigue Results

#### 2.5.1. Failure Modes

**Figure 9:**
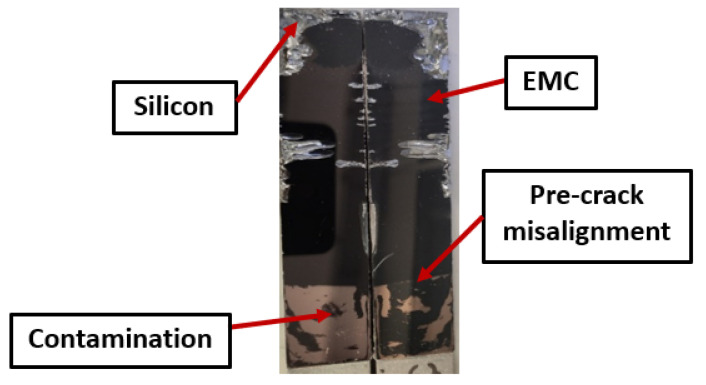
Typical fracture surface for type-two-interface fatigue testing.

In fatigue testing, only the type-two interface was examined, leading to only interfacial failure mode results. Figure 9 illustrates a typical fatigue fracture surface. Similar challenges, including pre-crack misalignments, contaminations, and edge irregularities, were observed in fatigue testing as reported in quasi-static testing.

#### 2.5.2. Fracture Analysis

**Figure 10:**
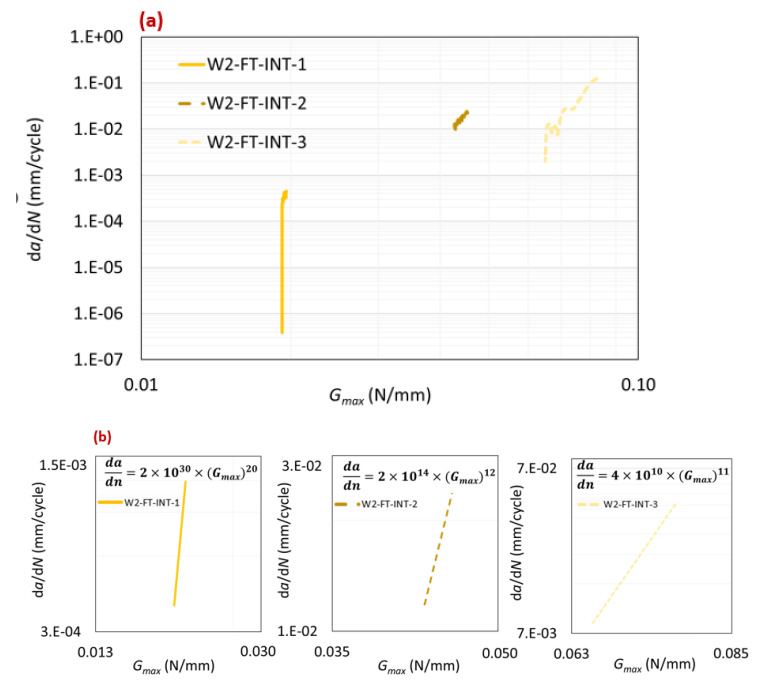
Paris law curves for type-two interface (**a**) and detailed view of stable crack propagation area (**b**).

**Figure 11:**
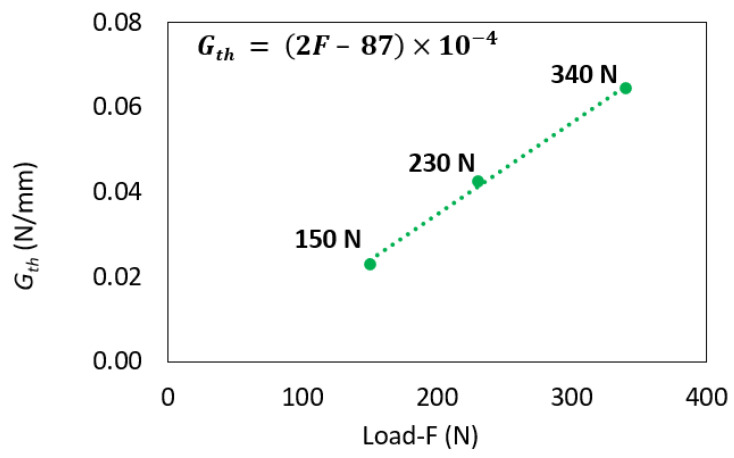
Linear relation between $G_{th}$ and applied load.

The average values obtained for *m* and for $G_{th}$ were 14.33 and 0.042 N/mm, respectively. Figure 10a presents a summary of the three obtained Paris law curves. Figure 10b provides a detailed zoom-in of the stable crack propagation area for each curve. Analyzing both figures revealed notable scatter between the curves. In the load control fatigue test, as the maximum load was reduced, the obtained $G_{th}$ decreased until it reached the true $G_{th}$ of the bi-material interface. The results from the fatigue tests indicated a linear relationship between $G_{th}$ and the applied maximum fatigue load, as depicted in Figure 11. Determining $G_{th}$ was crucial as it represents the minimum energy required to initiate a fatigue crack.

Table 4 categorizes and summarizes all of the obtained fatigue results.

**Figure 12:**
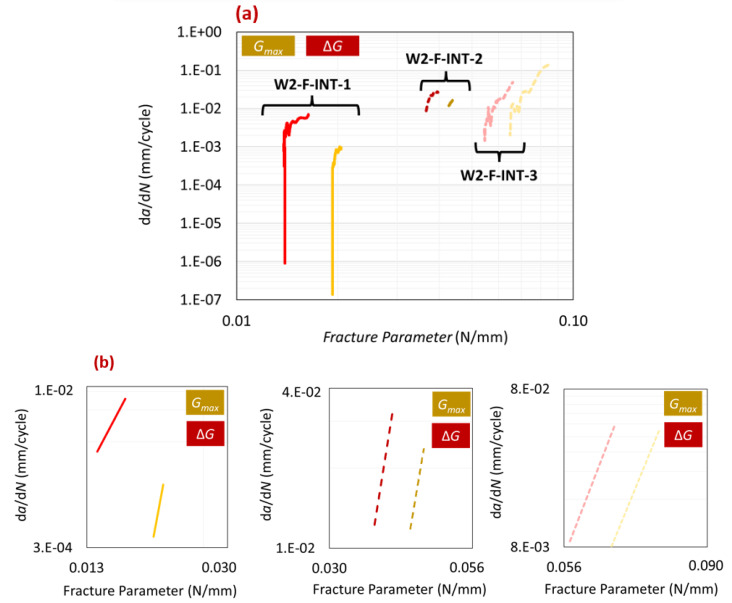
(**a**) Paris law curves when using fracture parameters ΔG and $G_{max}$. (**b**) Trendline comparison for the stable crack propagation region of the Paris law curve.

In the cyclic loading tests in this study, a constant R-ratio of 0.33 was maintained, while the maximum load was varied across different tests. To account for the effects of $G_{min}$, the Paris law relation was modified to use ΔG instead of $G_{max}$. Figure 12a compares the Paris law curves using both fracture parameters, while Figure 12b presents the trendlines of the stable crack propagation region for each test. Table 5 summarizes and compares the results of all tests using both fracture parameters. As is evident in Figure 12a, there was a leftward shift transitioning from the $G_{max}$ Paris law curve to the ΔG Paris law curve. This shift was attributed to the influence of $G_{min}$ as it decreased the values on the x-axis of the Paris law graph. Regarding the slope (*m*), it remained nearly unchanged, except in the case of test W2-FT-INT-1. Although not easily discernible in Figure 12, Table 5 highlights that using ΔG resulted in a slightly tighter range of *m* and $G_{th}$ values. These observations align with findings reported in the literature. Rocha et al. [19] and Erpolat et al. [20] have documented similar results, emphasizing a notable difference in $G_{th}$ values and a comparatively smaller difference for *m*.

### 2.6. Comparison with the Literature

**Figure 13:**
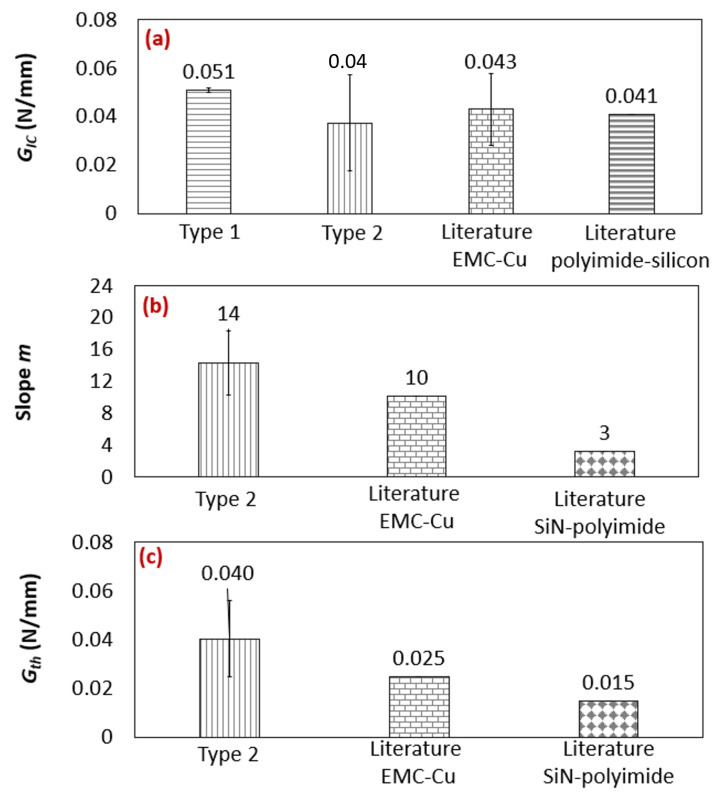
(**a**) $G_{IC}$ comparison with the Cu–EMC [2,3,4,5,21] and polyimide–silicon interfaces [7] from the literature and (**b**) *m* and (**c**) $G_{th}$ comparison with the Cu–EMC [2] and SiN–polyimide [7] interfaces from the literature.

In Figure 13a–c, the experimental results of this study are compared with the average values reported in the literature for $G_{IC}$, *m*, and $G_{th}$, respectively, across different types of bi-material interfaces.

For fatigue fracture test comparisons, only one paper was identified for each type of interface. In this study, ΔG was utilized as the fracture parameter in the Paris law relation. In the static fracture test, the average of all the values reported in the literature for the Cu–EMC interface is presented. For the Cu–EMC interface, the reported range of $G_{IC}$ values in the literature was from 0.036 to 0.060 N/mm. It is important to note that polyimide–silicon has only been analyzed in one study. The range of values for each parameter obtained in this study can be observed in Table 3 and Table 4. When comparing the results of this study to the literature, the values of the type-one interface were the highest, while the type-two-interface values were the lowest.

## 3. Conclusions

Silicon and EMC, widely used in the semiconductor field, lack comprehensive research on their behavior as a pair. This study focused on the quasi-static and cyclic loading behavior of silicon–EMC interfaces and faced challenges in producing thin films with pre-cracks and dealing with silicon’s fragile nature. Despite these hurdles, the study successfully obtained satisfactory results, providing insights into the bi-material interface for this material pair.

Various quasi-static and fatigue parameter values were determined using CBBM. The mode I fracture energy for type-one and type-two interfaces was found to be 0.05 N/mm and 0.04 N/mm, respectively. For fatigue, the type-two interface exhibited a fatigue threshold energy of 0.042 N/mm and *m* of 14.33. Considering the effects of $G_{min}$ with ΔG instead of $G_{max}$ resulted in a slightly tighter range of values for the fatigue threshold energy, with no significant changes in *m*.

The findings offer valuable insights into chip packaging failure modes and pave the way for future research on material properties and the impact of manufacturing parameters and temperature conditions on multilayer semiconductor delamination. It is also crucial to consider the influence of residual stresses during wafer assembly fabrication as a subject for future investigation.

## Figures and Tables

**Table 1:** List of material properties [14,15].

Materials	Ultimate Tensile Strength (MPa)	Poisson’s Ratio	Young’s Modulus (GPa)
Silicon	165	0.28	112
EMC	90	0.38	2.36
PM300	1020	0.33	205
Polyimide	300	0.4	3.73
Silicon oxide	45	0.17	73

**Table 2:** AV138 adhesive properties determined with thick adherend test [16,17].

Young´s modulus (GPa)	4.59 ± 0.81
Tensile yield strength (MPa)	36.49 ± 2.47
Ultimate tensile strength (MPa)	41.01 ± 7.28
Tensile failure strain (%)	7.80 ± 0.70
Poisson’s ratio	0.35

**Table 3:** Quasi-static results for different types of interfaces and failure modes.

Test Code	Interface Type	Failure Mechanism	Max Load (N)	Max Displacement (mm)	$G_{\mathrm{IC}}$ (N/mm)
W1-ST-INT1	1	Interfacial	292	0.456	0.052
W1-ST-INT2			260	0.454	0.050
W2-ST-INT-1	2		210	0.219	0.028
W2-ST-INT-2			184	0.292	0.019
W2-ST-INT-3			270	0.483	0.065
W1-ST-MIX-1	1	Mixed	574	0.653	0.155
W1-ST-MIX-2			321	0.561	0.065
W1-ST-MIX-3			317	0.611	0.075
W1-ST-MIX-4			371	0.626	0.045
W2-ST-MIX-1	2		188	0.369	0.03
W2-ST-MIX-2			194	0.192	0.035
W2-ST-SIL-1	2	Silicon	431	0.857	0.125
W2-ST-SIL-2			225	0.551	0.095

**Table 4:** Summary of fatigue results.

Test Code	Interface Type	Failure Mechanism	Fatigue Load (N)	FCG Rate (mm)	$G_{\mathrm{th}}$ (N/mm)
W2-FT-INT-1	Two	Interfacial	150	20	0.0192
W2-FT-INT-2			230	11	0.0427
W2-FT-INT-3			340	11	0.0645

**Table 5:** Results of analyzing each test with a different fracture parameter.

Fracture Parameter	Test Code	Slope, *m*	$G_{\mathrm{th}}$ (N/mm)
$G_{max}$	W2-FT-INT-1	20	0.0192
	W2-FT-INT-2	11	0.0427
	W2-FT-INT-3	11	0.0645
ΔG	W2-FT-INT-1	7	0.0138
	W2-FT-INT-2	12	0.0367
	W2-FT-INT-3	11	0.0546

## Data Availability

Data are included in the article.
